# New Horizons in Enhancing the Proliferation and Differentiation of Neural Stem Cells Using Stimulatory Effects of the Short Time Exposure to Radiofrequency Radiation

**Published:** 2015-09-01

**Authors:** M. Eghlidospour, S. M. J. Mortazavi, F. Yousefi, S. A. R. Mortazavi

**Affiliations:** 1 Medical Physics and Medical Engineering Department, School of Medicine, Shiraz University of Medical Sciences, Shiraz, Iran; 2 Ionizing and Non-ionizing Radiation Protection Research Center (INIRPRC), Shiraz University of Medical Sciences, Shiraz, Iran; 3 Medical Student, Student Research Committee, School of Medicine, Shiraz University of Medical Sciences, Shiraz, Iran

**Keywords:** Stem cell, Proliferation, Differentiation, Radiofrequency (RF), Diseases

## Abstract

Mobile phone use and wireless communication technology have grown explosively over the past decades. This rapid growth has caused widespread global concern about the potential detrimental effects of this technology on human health. Stem cells generate specialized cell types of the tissue in which they reside through normal differentiation pathways. Considering the undeniable importance of stem cells in modern medicine, numerous studies have been performed on the effects of ionizing and non-ionizing radiation on cellular processes such as: proliferation, differentiation, cell cycle and DNA repair processes. We have conducted extensive studies on beneficial (stimulatory) or detrimental biological effects of exposure to different sources of electromagnetic fields such as mobile phones, mobile phone base stations, mobile phone jammers, radar systems, magnetic resonance imaging (MRI) systems and dentistry cavitrons over the past years. In this article, recent studies on the biological effects of non-ionizing electromagnetic radiation in the range of radiofrequency (RF) on some important features of stem cells such as their proliferation and differentiation are reviewed. Studies reviewed in this paper indicate that the stimulatory or inhibitory effects of RF radiation on the proliferation and differentiation of stem cells depend on various factors such as the biological systems, experiment conditions, the frequency and intensity of RF and the duration of exposure.

## Introduction

The electromagnetic spectrum is composed of frequency ranges from 10 Hz to 10^24^ Hz and wavelength from 10^(-16)^ to 10^7^. Based on the amplitude and frequency band, the electromagnetic spectrum is divided into two main categories: ionizing and non-ionizing radiation[1]. The interaction of ionizing radiation with biological material causes excitation or ionization of its atoms by transferring a large amount of energy in a very limited area. X-rays, gamma, beta and alpha are ionizing radiation [2]. Rays that pass through the material and are not able to cause ionization but have enough energy to excite are called non ionizing radiation, while that is despite the fact that some biological damages caused by non ionizing radiation are known. The non-ionizing radiation spectrum is divided into optical (light) and electromagnetic radiation. Optical radiation includes ultraviolet, visible and infrared light and radio waves. Radio waves include high frequency microwave and low-frequency radio emission [3]. Microwaves are at a frequency range of 300 MHz to 300 GHz.  Microwave and radio frequency radiation sources include radar systems for air traffic and weather reports, personal communication systems, telephone equipment for long-distance, microwave ovens, industrial machinery, medical treatment and diagnostic equipment, and medical diathermy devices [4]. 

## Biological effects of microwave radiation

Biological effects of microwave radiation are divided into two categories: thermal and non-thermal effects.

Thermal effects of radio frequency are primarily related to absorbed energy and lead to electrical conductivity in most tissues. The other factor is the creation of a non-free rotation of molecules, particularly water molecules. And this work is done by transferring energy to the liquid, and finally this energy presents as heat [5]. RF  radiation can penetrate into the tissues of the organism, absorb and convert to heat. Energy absorption and distribution of the EMF in an organism depends on several factors such as: the combination of the dielectric irradiated tissue, the frequency of EMF, shape, geometry and distance from the source, the intensity of EMF, duration, and stage of development [6]. Since the energy per photon is generated by RF radiation is not strong enough to cause chemical changes directly in the cell, Biological effects induced by RF radiation are indirectly, which make possible changes in cell function such as: Inhibiting the synthesis, transcription, processing of DNA, translation of RNA, progression of cell cycle, and changes in cell metabolism and cell membrane permeability. ROS  induced by oxidative stress resulting from the emission of RF is considered as an important factor in tissue damages such as: lipids, proteins, and DNA degeneration [7, 8]. During the past several years, Mortazavi et al. at Shiraz University of Medical Sciences have done extensive studies on the beneficial or stimulation and deleterious biological effects of exposure to electromagnetic fields Such as: mobile phones and mobile phone base stations [9-14], mobile phone jammer [15], radar systems [16], dental cavitrons [17], and magnetic resonance imaging system [18]. About the beneficial and stimulating effects of electromagnetic field of RF radiation, studies on the adaptive response have been done by Mortazavi et al. in the years 2011-2013 [9, 13, 19]. In the other studies, improvement in cognitive function in humans [4, 20] and animals [21] are shown. Mortazavi et al in a recent study has reported the beneficial cognitive effects after short-term exposure of human to radiation of mobile phones. The report, which published in 2011, Mortazavi et al. showed that Visual reaction time (VRT) of students significantly reduced after 10 minutes exposure to the EMF of cell phone [22]. They concluded that this radiation reduces the reaction time, which leads to a better response to environmental risks. They also showed that occupational radiation exposure of radar systems reduces the reaction time of radar workers [16]. In other words, positive cognitive effects of prolonged exposure to high-frequency EMF have been found in some epidemiological studies, showed that high-frequency EMF radiation improves cognitive behavior in laboratory animals. In this regard, the increase in brain glucose consumption after exposure to RF radiation has been confirmed by PET studies that could be a potential mechanism for this phenomenon [23]. In another study by using an interference test, Arns et al. (2007) showed that prolonged use of cell phones improves the performance in normal subjects [24]. Also the beneficial effects of electromagnetic fields on neural degeneration diseases such as Alzheimer’s disease, Schulzet al. (2009) demonstrated that long-term use of mobile phones reduces the risk of Alzheimer’s disease about 30-40 percent [25, 26]. 

## The effects of microwave radiation on the central nervous system

Global use of mobile phones in recent decades has been an explosive increase, which leads to concern about the possible biological effects of this technology on human health. Electromagnetic fields emitted by mobile phones include frequency range from 800 to 2000 MHz, which are known as radio frequency waves. According to the global system of mobile communication (GSM), frequency bands of 900 and 1800 MHz are of the most widely used. However, the biological effects of EMF of cell phones on mammalian cells in many respects are unknown [27, 28]. Placement of the cell phone antennas near the head and scattering the waves, which generated by base stations in the surrounding environment, cause the propagation of radio wave near the brain and nervous system [29].

In single cell organisms, MW / RF radiation induces damages that can lead to death, preventing proliferation, DNA damage and changes in gene expression in various cell types including brain cells [30]. In a study using Immunohistochemistry method, it has been shown that radio waves GSM 900 MHz with SAR = 6 w / kg increase the level of gliogenesis in the striatum and cortex of rat brain [31]. In another study exposure to GSM 900 MHz with SAR = 6 w / kg has shown a significant reduction in CO activity in some brain areas. The results of this study have shown that radio waves can affect neural activity or brain metabolism in rats [32]. In another study which was conducted on pregnant rats under different intensities of mobile phone radio waves for assessing the level of neurotransmitters and oxidative stress in the brain of rats and measuring the value of  (SOD) (Super dismutase), (GSH-PX) (Glutathione peroxidase), (MDA) (Malondialdehyde), (NE) (Noradrenaline), (5-HT) (5-hydroxyindole acetic acid), was shown that exposure to mobile phone radio waves during pregnancy can induce detrimental effects on fetal brain [33]. The underlying mechanism may be due to increased production of ROS, defects and damages in mitochondrial activity, disruption of the intracellular calcium homeostasis, protein expression in response to temperatures higher than normal, and alteration of the specific gene expression in the brain [33-37]. Epidemiological and laboratory animal studies have reported that the EMF of cell phones causes neurological behavior disorders, loss of elasticity in the neurons of the hippocampus, increased permeability of the blood-brain barrier, and higher risk of degenerative diseases and brain tumors [38-41]. However, the effects of EMF of radio waves on brain development remain largely unknown. Currently, more information about the effects of EMF on brain has been attained from primary neural cultures and tumors or immortalized cell lines. However, extrapolating these results to in vivo situations is not always possible, but the stem cell technology provides a new approach to better understand the responses and reactions induced by environmental hazard [42]. Stem cell technology is particularly useful in developmental neurotoxicology research, because they are widely available in developmental systems [43]. 

## Introducing stem cells

Stem cells are unspecialized cells in the human body, which have the ability to become specialized cells with specific functions. As a rule, a stem cell remains uncommitted until getting a command signal to develop into specialized cells. Stem cells can be regarded as a repair system, which are able to divide unlimitedly to replace the other cells. When a stem cell divides, each new derived cell has the potential either to remain a stem cell or become a new specialized cell such as brain or blood cells. Most of the repair procedures in mammals are independent differentiation events, which are created by the activation of the stem cells or precursors in tissues.

Stem cells, according to their source, are classified into four types: embryonic stem cells, fetal stem cells, umbilical cord stem cells, and adult stem cells that each of them has their own sub-groups. Fetal and adult stem cells derive from embryonic stem cells. However a small number of stem cells in adult organs are the remainders of embryonic stem cells, which have remained in the race of differentiation to the developing organs and are left in the cell niches until they are called for repairing damaged tissues. Embryonic stem cells include neural stem cells, hematopoietic stem cells, and pancreatic islet precursor. Embryonic neural stem cells in the fetal brain have the ability to differentiate into neurons and glial cells. Adult stem cells include hematopoietic stem cells (bone marrow or peripheral blood cells), mesenchymal stem cells (bone marrow stromal cells), gastrointestinal tract stem cells, liver stem cells, cartilage and bone stem cells, epidermis (skin) stem cells, pancreatic stem cells, and neural stem cells [44, 45]. 

## Neural stem cell

Development of mammalian brain during fetal period starts from neuroepithelial cells around the primary ventricular zone. In this area the cells divide actively and participate in the formation of other brain structures [46]. Researchers have found that the population of precursor cells in the adult brains multiplies continuously, which not only have the ability of self-renewing but also generate a large group of central nervous system cells such as neurons, astrocytes, and oligodendrocytes [47]. Generally, the areas of adult brain, which their neurogenesis has been done, are: amygdala, cerebellum, cerebral cortex, sub ventricular zone (SVZ), sub granular zone (SGZ), and the dentate gyrus [48]. The sub ventricular area is more active than other areas, because its cells multiply too much during restoration of lesions and degenerative changes [49]. This area, during embryonic development, after forming the ventricular zone, creates a secondary germinal layer in the ventricular zone, which is called the sub ventricular zone. This area is depleted after birth and remains as a thin layer of sub ventricular zone, which creates adult SVZ [50]. The electron microscopy structure of adult SVZ shows that this area has ependymal and three types of cells, B, A and C. Type B cells are neural stem cells (NSC), which in addition to itself renewal, create type C cell which is said (TAC) [51]. Dividing of type C cell creates type A cells, which are known as neuroblasts; after moving to different areas of the track including (RMS), they reach into the olfactory Bulb areas and become mature neural cell types. Type B cells that are like astrocytes and express GFAP factory, have two types. B cells have rich microenvironment with access to the cerebro-spinal fluid (CSF) within the ventricles, the expand blood vessels, ependymal and epithelial root factors [53]. Using a combination of surface markers, true stem cells can be isolated and purified. The most common method for the isolation and expansion of these cells is cultured neurosphere assay (NSA). In this method, the precursor and neural stem cells, which are able to clonal proliferation from colonial cells called neurosphere in the culture [52]. 

## Biological effects of microwave radiation on stem cells

Given the importance of stem cells, studies on the effects of ionizing and non-ionizing radiation on cellular processes such as proliferation, differentiation, cell cycle, DNA repair processes have been carried out. For example, one study  showed that the dose rate of proton radiation on neural stem cells in mice and humans can affect the level of oxygen activated species and nitrogen activated species [54]. In another study, with the aim of evaluating the effects of proton radiation on mouse neural stem cell, have been shown that Exposure to ionizing radiation of protons at a range of dose between1-10 Gy increases the level of activated oxygen species [55]. In another study, it was shown that low doses of ionizing radiation can easily affect apoptotic processes in human embryonic stem cells, which this effect depends on the dose, dose rate, linear energy transfer (LET), and other characteristics of the microenvironment [56]. About non-ionizing radiation studies on stem cells, Kanda A et al showed that extremely low frequency electromagnetic fields may change cellular process by increasing the concentration of intracellular reactive oxygen species in human mesenchymal stem cells. Producing of Reactive oxygen species, is not limited to phagocytic cells, but also includes the processes of differentiation and proliferation of mesenchymal stem cells [57]. In another study that was conducted in 2012 by Cho et al, demonstrated that radiation of electromagnetic fields induces neural differentiation of mesenchymal stem cells [58]. In another study for appraising the effect of mechanism and the induction of neural differentiation of mesenchymal stem cells, Jeong-Eun Park et al showed that the electromagnetic fields with increasing EGFR signals and very low rate of reactive oxygen speciesproduction, cause neural differentiation of mesenchymal stem cells [59]. 

## The effects of microwave radiation on neural stem cells

Due to the widespread use of mobile communication systems in daily routine life, determining the effect of EMF-RF radiation on the brain evolution is a major concern, because any environmental stimulation during brain development that can affect the fate of neural stem cells may disrupt brain development.

Embryonic neural stem cells are considered pluripotent cells because they can differentiate and be divided into three main categories of brain cells [60]. These cells can be derived from the fetal nervous system tissues. The fate of embryonic neural stem cells in the brain evolution is very important. This process involves proliferation and ddifferentiation into glial and neural cells, cell death and the development of neurite. Previous studies have revealed that exposure of rodents to EMF-RF may lead to amendment of cells. This effect depends on many variables including specific absorption rate (SAR) of target and duration of exposure. The parameter SAR specifies the energy that a tissue absorbs per unit of mass that is expressed in W / kg and is used widely to measure the EMF-RF radiation dose [61]. A study found that repeated exposure to radiation of GSM 900 MHz with SAR = 2 W/kg causes the loss of neurons in the brain of rats in uterus [62]. In another study, increased level of transcription gene in embryonic neural stem cells, has been seen after exposure to EMF- RF radiation from GSM 1710 MHz SAR = 1.5W / kg [63]. In another study the changes in neural activity during the developmental phases were observed after exposure to EMF-RF 1800-900 MHz SAR=1.6 w/kg [64]. Determining the effects of EMF-RF radiation on proliferation, apoptosis of embryonic neural stem cells and gene expression related to apoptosis (Caspase-3, Bax, Bcl-2) is important. Many previous studies on cell lines and animal models show no effect of EMF-RF on proliferation, gene expression and apoptosis [65], while many other reports demonstrated the loss of neurons in the brain and also increase of the genes-related apoptosis in embryonic neural stem cells after exposure to EMF-RF radiation [66]. Another study has shown that GADD45 MRNA level in neural stem cells, is increased by exposure to EMF-RF. In this study, there was no change in cell cycle [63]. This inconsistency may be due to different cell models, SAR radiation, duration and frequency of radiation exposure to the source. Another study with the aim of evaluation of the high-intensity pulsed electromagnetic fields effect on proliferation and differentiation of neural stem cells in newborn rats with a frequency of 0.1 Hz and with an intensity range from 0.5 to 5 Tesla, found that exposure to electromagnetic fields can affect the proliferation of neural stem cells within a certain intensity (0.4 Tesla). It reported an increase in proliferation and growth of neural stem cells, while in the intensity range6-10 Tesla, a reduction in the growth and proliferation of neural stem cells has been reported. The results of differentiation, showed no significant change in the percentage of stem cells differentiated into neurons or astrocytes [67]. According to a new study in 2014 with the effects of 1800 MHz radio wave on neural stem cells, it was shown that radio waves will not make a difference in death, proliferation and rate of differentiation into neurons and astrocytes of embryonic neural stem cells. However, in this study it was shown that 1800 MHz EMF-RF radiation causes damage and impairment in expression of the helix-loop-helix (bHLH) that will be essential for neuronal development [68]. The effects of EMF radiation on proliferation of adult neural stem cells are widely used in very low frequency (ELF) and shows that ELF-EMF exposure causes an increase in the proliferation and differentiation of neural stem cells [69-72]. All results definitely show that the EMF-RF may affect various processes of neural stem cells’ evolution.

Studies conducted in this paper indicate that the stimulatory or inhibitory effects of radio waves on the proliferation and differentiation of stem cells depend on several factors such as biological systems, test conditions, frequency, intensity and duration of exposure. Development of therapeutic applications of stem cells in the restoration of lesions and neurological changes has increased the need for research in this area.  You can also learn more about issues related to positive and stimulatory effects of radio waves of cell phone on the proliferation and differentiation and use this technology to accelerate the proliferation and differentiation of stem cells or on the opposite side with recognizing the harmful effects of radiation on stem cells, and prevents the disruption in the stem cell therapy. 
